# The Role of Information in Dental Traumatology in Patients during Developmental Age: A Cognitive Investigation

**DOI:** 10.1055/s-0041-1735789

**Published:** 2021-10-22

**Authors:** Francesco Saverio Ludovichetti, Anna Giulia Signoriello, Andrea Zuccon, Sharon Padovani, Sergio Mazzoleni

**Affiliations:** 1 Department of Neurosciences, Dentistry Section, Università degli Studi di Padova, Padova, Italy

**Keywords:** dental trauma, developmental age, knowledge

## Abstract

**Objectives** The gestation period involves a series of changes in all maternal organs and systems, due to hormonal fluctuations that also affect the oral cavity, leading to possible development of diseases such as gingivitis, oral pregnancy tumor, or periodontitis. Over the years, the positive correlation between poor oral health and adverse outcomes in pregnancy, such as fetal changes, low birth weight, preterm birth, or preeclampsia, has also been highlighted. The aim of this study is to analyze and understand the levels of knowledge and information of pregnant women about the possible repercussions that they may have on the oral cavity, caused by hormonal alterations.

**Materials and Methods** A questionnaire of 17 multiple choice questions was formulated and published online from 01/02/2020 to 31/08/2020, aimed at pregnant women and new mothers. The Google Forms platform was used to collect the data that were then statistically analyzed by creating crosstabs with multiple dependency variables.

**Results** The study cohort was represented by 1,191 women, whose responses first showed that about half were unaware of the predisposition to develop infections and periodontal problems during the gestation period. The same noticed bleeding and gum swelling during brushing and despite this, only 21% visited their dentist to resolve the situation. Furthermore, 88.2% of all women did not know that problems in the oral cavity can lead to adverse pregnancy outcomes.

**Conclusion** Primary prevention is essential and must be implemented with the collaboration of the various professional figures who follow the woman during gestation.

## Introduction

A dental trauma is the consequence of an accidental event with damage to the hard and supporting structures of the tooth. It can be “direct” when the damage directly involves the orofacial area, or “indirect” when the orofacial area is affected as a consequence of another type of trauma. 1 2 As far as the mechanisms of action and some variables are concerned, such as the force and the direction of the impact and the nature of the traumatic object, the severity of the damage may be different. 1 3

Due to the high frequency of accidental events and to the complexity of the intervention and costs, dental trauma is considered a real dental emergency.

It is estimated that the incidence is ∼30% in deciduous teeth and 20% in permanent ones. 2

Furthermore, it can cause both aesthetic and functional problems, with a possible impact on the patient's quality of life; the most affected elements are, in fact, the anterior incisors, which play a fundamental role in chewing, in the integrity of the supporting tissues and in phonetics. 4

From the etiological point of view, there are several predisposing risk factors:

- At the anatomical level, the presence of an overjet > 3.00/3.5 mm, associated with dental and skeletal malocclusions and with situations of labial incompetence, leads to a greater exposure of the elements 1 5 6 ;
- At the demographic level, the most affected subjects are children aged ⅔, due to lack of coordination in the early stages of walking, and 8/10 years old due to sports activities with a prevalence in males 1 2 3 4 5 ;
- At the anthropometric level, the physical and nutritional state play an important role, as obese subjects are characterized by less agility in movements 7 8 9 ;
- At an environmental level, places such as the house, where dangerous edges, stairs, school, especially in recreational moments, sports fields, gyms must be taken into consideration 1 5 6 8 9 ;
- At the behavioral level, the presence of bad habits. 1 6 9

Traumatic injuries can be limited to the dental elements, respectively the periodontium and the alveolar bone, or involve all these structures at the same time. 2 The diagnostic examination will involve the use of different types of radiographic techniques and both thermal and electrical tests on dentinal sensitivity. The goal of the treatment is to maintain the vitality of the pulp; therefore, an immediate and appropriate intervention is essential. 10 Knowledge of emergency treatment is not only the responsibility of the dentist but also of those who live or work with children and adolescents, such as parents, teachers, and sports coaches.

Unfortunately, the level of information and knowledge is often limited, and methods such as washing or a correct conservation of an avulsed or fractured element in the period between the trauma and the specialist visit are not sufficient. This leads to an increase in poor prognosis. 11

The main purpose of this study was to investigate the knowledge of parents, guardians, teachers, sports coaches about dental trauma in children and adolescents.

From the statistical evaluation of the data obtained, the final goal is to obtain a general picture of the perception of this dental problem, with the aim of providing the information that is necessary for a correct and immediate approach to emergency.

## Materials and Methods

A total convenient sample of 1,191 people was included in the study; all the people who were permanent resident in Italy, who were parents, guardians, teachers and sports coaches of children and adolescents from 0 to 16 years and gave the consent of participation in the research, were included in the study. Drafting, creation, and collection processes were performed at the Department of Pediatric Dentistry of the Borgo Cavalli clinic in Treviso (University of Padova).

Data collection was performed from January 24, 2020 to September 3, 2020, through the online publication of a questionnaire (free online platform of Google Forms). The questionnaire reliability was checked by conducting a pilot study on 30 parents, guardians, teachers, and sports coaches of children and adolescents from 0 to 16 years who were not a part of the final study sample.

Before starting data collection, each patient signed a Free and Informed Online Consent Form, consisting of a page explaining the research, in addition to the request for authorization to use the data. All participants were guaranteed complete confidentiality of information and the possibility of not participating in the research if they did not wish to. Furthermore, the purpose and the importance of the research were stressed to all.

Each parent answered an online questionnaire with Appendix A .

## Results

### Chi-Squared Test

The chi-squared (χ ^2^ ) independence test is used to verify the existence of an association between the variables of the contingency table built from the sample data. The null hypothesis (H0) represents the fact that the variables are not associated and independent. On the other hand, the alternative hypothesis (H1) confirms that the variables are associated or dependent.

In Tables 1 , 2 , 3 , and 4 , it is possible to find the frequency according to the age variable: all children under 6 were classified as “preschool,” while the remaining, those who were 6 years or older, were considered to be of “school age.”

**Table 1:** Frequency distribution of responses according to school age

Are you aware of the fact that, in the event of a fracture and detachment of a dental fragment, it should NOT be dried with paper and stored in foil, but must be kept moistened to avoid necrosis of the tissue fibers?	Age	
	Preschool age (%)	School age (%)
No	229 (24.36)	393 (41.81)
Yes	80 (9.47)	229 (24.36)

Note: Chi-squared test = 6.9388, df = 1, *p* -Value = 0.0084.

**Table 2:** Frequency distribution of responses according to school age

What is the maximum time limit within which to go to a dental office?	Età	
	Preschool age (%)	School age (%)
6 h	87 (9.26)	153 (16.28)
12 h	67 (7.13)	103 (10.96)
24 h	164 (17.45)	366 (38.94)

Chi-squared test = 5.9668 df = 2, *p* -Value = 0.0258.

**Table 3:** Frequency distribution of responses according to school age

If the tooth has completely come out of the bone (avulsion), do you know that you should try to re-implant the tooth in the socket or, if this is not possible, store it in milk or saline and go to the dentist within 60 minutes to have a favorable prognosis?	Age	
	Preschool age (%)	School age (%)
No	227 (24.15)	410 (43.62)
Yes	91 (9.68)	212 (22.55)

Note: Chi-squared test = 2.6345, df = 1, *p* -Value = 0.1046.

**Table 4:** Frequency distribution of responses according to school age

Do you know that in the 10/15 days following the trauma a soft diet is recommended and, in younger children, the use of a pacifier is not recommended?	Age	
	Pre-school age (%)	School age (%)
No	143 (15.21)	252 (26.81)
Yes	175 (18.62)	370 (39.36)

Note: Chi-squared test = 0.7798, df = 1, *p* -Value = 0.2153

Table 1 summarizes the answers to the following question: “Are you aware of the fact that, in case of fracture and detachment of a dental fragment, it should NOT be dried with paper and stored in film, but kept moist to avoid necrosis of the tissue fibers?” It is possible to observe that only 33.83% of parents knew the procedures in case of a fracture or detachment of a tooth fragment. Moreover, it is noted that of the 34% mentioned before, 24.36% had children of school age, while 9.47% attended kindergarten.

Table 2 analyzes the variable “What is the maximum time limit within which to go to a dental office” (in the event of a fracture or detachment of a dental element) based on the age group. The majority of parents (38.94%) answered “24 hours” and had their own children of school age. It can be seen that the *p* -value of the test calculated in the table was less than 0.05.

As in the previous results, the null hypothesis is rejected and there is an influence of the school age on the parents' response regarding the time limit for taking their child to a dental office in the event of a fracture or loss of a dental element.

In Table 3 , we study the variable “If, on the other hand, there is a complete escape of the tooth from the bone (avulsion), you know that you should try to re-implant the tooth in the alveolus or, if this is not possible, store it in milk or saline and go to the dentist within 60 minutes to have a favorable prognosis?” according to the age group.

The majority of parents (67.77%) was not aware of the procedures in case of tooth avulsion: this result is worrying because this lack of knowledge can influence the final prognosis.

The *p* -value of the test calculated in the table was greater than 0.05. Unlike the previous results, the null hypothesis will not be rejected, that is, there is no influence of school age on the parental response in this case.

Table 4 shows the analysis of the variable “Do you know that a soft diet is recommended in the 10/15 days following the trauma and, in younger children, the use of a pacifier is not recommended?” depending on the age group. The majority of parents (57.98%) are aware of this. We can see that the *p* -value of the test calculated in the table was greater than 0.05. As in the previous result, the null hypothesis will not be rejected: there is no influence of school age on the parental response in this case.

In Table 5 , we investigate the variables “Are you aware of the fact that, in case of fracture and detachment of a dental fragment, it should not be dried with paper and stored in foil, but kept moist to avoid necrosis of the tissue fibers?” and “Have you ever heard of” dental trauma “?” About 50.64% of respondents have never heard of dental trauma and their parents are unaware of the fracture and detachment procedure, which is worrying. Furthermore, as far as the parents who have already heard of dental trauma are concerned, only 40% knew what to do in this type of situation. The *p* -value is lower than 0.05 and, therefore, the null hypothesis is rejected: hearing about dental trauma influences the response of parents regarding the knowledge of the procedures to be applied.

**Table 5:** Frequency distribution of responses according to dental trauma knowledge

Are you aware of the fact that, in the event of a fracture and detachment of a dental fragment, it should NOT be dried with paper and stored in foil, but must be kept moistened to avoid necrosis of the tissue fibers?	Have you ever heard of “dental trauma”	
	No	Yes
No	476 (50.64%)	146 (15.53%)
Yes	218 (23.19%)	100 (10.64%)

Note: Chi-squared test = 6.518, df = 1, *p* -Value = 0.0107.

Table 6 contains the variables “What is the maximum time limit within which to go to a dental office?” and “Have you ever heard of dental trauma?” It is possible to notice that most of the parents answered 24 hours as the maximum time.

**Table 6:** Frequency distribution of responses according to dental trauma knowledge

What is the maximum time limit within which to go to a dental office?	Have you ever heard of “dental trauma”	
	No (%)	Yes (%)
6 h	175 (18.62)	65 (6.91)
12 h	111 (11.61)	59 (6.28)
24 h	408 (43.40)	122 (12.98)

Note: Chi-squared test = 9.2381, df = 2, *p* -Value = 0.0099.

As far as the *p* -value is concerned, a result of less than 0.05 is found and therefore the null hypothesis is rejected.

In Table 7 , we consider the variables “If, on the other hand, there is the complete escape of the tooth from the bone (avulsion), do you know that you should try to re-implant the tooth in the alveolus or, if this is not possible, store it in milk or physiological solution and go to the dentist within 60 minutes to have a favorable prognosis?” and “Have you ever heard of dental trauma?”

**Table 7:** Frequency distribution of responses according to dental trauma knowledge

If the tooth has completely come out of the bone (avulsion), do you know that you should try to reimplant the tooth in the socket or, if within 60 minutes to have a favorable prognosis?	Have you ever heard of “dental trauma”	
	No (%)	Yes (%)
No	486 (51.70)	151 (16.06)
Yes	208 (22.13)	95 (10.11)

Note: Chi-squared test =5.8289, df = 1, *p* -Value = 0.0158.

It emerges that the large number of parents (67.76%) did not know how to correctly act when the event described in the question occurs, and among them 16% had already heard of dental trauma. This represents a problem because it shows that there is an information gap to make an informed decision. On the other hand, 10% of parents who have already heard of dental trauma knew that they should try to replant the tooth or store it in milk or saline and go to the dentist within 60 minutes to get a favorable prognosis. The *p* -value was less than 0.05 and therefore the fact that dental trauma has already been heard influences parents' knowledge on how to proceed in the situation described in the question.

Table 8 connects the variables “Do you know that a soft diet is recommended in the 10/15 days following trauma and, in younger children, the use of a pacifier is not recommended” and “Have you ever heard of” dental trauma “?” It is possible to notice that 15.43% of parents who had already heard of dental trauma knew the information contained in the request. Another positive point is that 42.55% of parents who have never heard of dental trauma also knew they have a light diet or use a pacifier (in young children) following the trauma. As far as the *p* -value is concerned, it is less than 0.05 and, therefore, having already heard of dental trauma positively affects the knowledge of the answer.

**Table 8:** Frequency distribution of responses according to dental trauma knowledge

Do you know that in the 10/15 days following the trauma a soft diet is recommended and, in younger children, the use of a pacifier is not recommended?	Have you ever heard of “dental trauma”	
	No (%)	Yes (%)
No	294 (31.28)	101 (10.74)
Yes	400 (42.55)	145 (15.43)

Note: Chi-squared test = 94.301, df = 1, *p* -Value = 2.2e-16.

## Discussion

A dental trauma is the consequence of an accidental event damaging the supporting structures of the tooth. 1

It can cause both aesthetic and functional problems, with a possible impact on the patient's quality of life; the elements most affected are, in fact, the incisors, which play a fundamental role in chewing, in the integrity of the supporting tissues, and in phonetics. 4

It is estimated that the incidence is ∼30% in the deciduous dentition and 20% in the permanent dentition. 2

Promptness and knowledge are fundamental in the treatment. They are characteristics of responsibility not only of the dentist but also of those who live or work with children and adolescents. 10 11

The purpose of this study is to investigate the knowledge of parents, guardians, teachers, and sports coaches about dental trauma in children and adolescents, to understand the perception of this problem, and to provide the information necessary for a correct and immediate emergency approach.

The sample of participants in the study consists of 1,191 subjects, all with at least one child between 0 and 16 years of age, of which 53.5% male and 46.5% female, and originating from 99% Italian.

As far as the question on the knowledge of the term “dental trauma” is concerned, 76.6% replied that they have already heard of it before, while 23.4% did not. Investigating the clinical history, it emerged that 27.4% previously reported a dental trauma, while 72.6% did not.

The knowledge about an individual predisposition and a higher incidence of trauma is not present in 63.3% and 78.4% of parents who, however, for 57.2%, are aware of the fact that damage to the deciduous dentition can cause damage even to the permanent dentition.

The feedback on possible dental necrosis following trauma in an immature tooth was positive (80.8%); also, it was positive regarding the greater impact of trauma on central incisors (87.7%) compared with lateral incisors. (5.9%) and canines (6.5%).

However, it is noted that 48.8% of the sample is not aware of which ages are most at risk of children and adolescents.

About 64.4% of the sample are unaware that they are keeping the tooth fragment moist; 53.2% believe it is right to keep it in water, 34% in milk, and only 12.8% in physiological solution.

About 56.3% would go to the dentist within 6 hours, 23.9% within 12 hours, and 19.8% within 24 hours.

In the event of an avulsion, 66.7% of the sample does not know what to do.

The indications of hygiene and nutrition are, however, clear respectively at 69 and 58.9%.

As far as the statistical analysis is concerned, the presence of an association between the variables obtained from the questions in the questionnaire was evaluated through the “Chi-Square” test.

Eight statistical tables were obtained on the basis of four questions taken twice, but compared with different variables, namely:

- The first 4 associated with the “age” variable, where all children under 6 were classified as “pre-school age,” while those who were 6 years or older were defined as “school age”;
- The second 4 related to a “previous knowledge” about dental trauma.

In Tables 1 and 5 , we have examined and connected to the two variables the question “Are you aware of the fact that, in the event of a fracture and detachment of a dental fragment, it should NOT be dried with paper and stored in foil, but rather kept moist to avoid tissue fiber necrosis?,” as cited in the literature. 5 6 10 12

In the first case, it is clear that only 33.83% of the sample was aware of the correct procedures for the conservation of a dental fragment and that, among the 34% who did not know, 24.36% interacted daily with children in school age. This data demonstrates the need for greater information between parents and teachers in contact with children in this age group. Games, discussions, bad habits (i.e., biting pen), sporting activities, including rugby, football, hockey, martial arts, basketball, volleyball, gymnastics, and outdoor activities that involve use of cycling, skateboarding, rollerblading, or skiing can increase the risk of dental trauma.

In these contexts, for example, preventive actions can be taken through the use of dental protections, aimed at reducing the effect of damage in the event of a struggle. 1 5 6 9 13 14

In the second case, it is worrying that 50.64% of those who have never heard of dental trauma and 40% of those who have already heard of it do not have the adequate knowledge to manage the emergency.

It is therefore clear the need of a large-scale information and in a targeted manner, through training courses aimed at parents, teachers, and facility staff, since even hearing about it does not necessarily lead to knowing a topic.

In Tables 2 and 6 , instead, we refer to the question “What is the maximum time limit within which to go to a dental office (in case of dental trauma)?”

The question is deliberately general to analyze the perception that people have about this emergency. The literature shows that it is necessary to go to a dental practice as soon as possible, no later than 2 to 3 hours after the event, to assess the extent of damage and increase the chances of favorable prognosis. The aim, in these cases, is to preserve the vitality of the cells and of the periodontal fibers. 6 13

Thus, in the analysis of Table 2 , it emerges that the majority of parents with children of school age, (38.94%) answered “24 hours,” a time limit much higher than that previously mentioned. The same answer also appears in the analysis of Table 6 .

The fact that a high percentage of the sample does not consider a dental trauma to the oral cavity as an emergency that requires an intervention as immediate as possible is worrying both on a practical and interventional and informative level. Considering the importance of timeliness in this circumstance, it is clear that the role played by primary prevention and preventive measures aimed at the protection of healthy subjects, through disclosure for information purposes. 3 5

In Tables 3 and 7 , the question is faced: “If, on the other hand, there is a complete escape of the tooth from the bone (avulsion), do you know that you should try to re-implant the tooth in the socket or, if this is not possible, physiological solution and go to the dentist within 60 minutes to have a favorable prognosis?”

Avulsion represents 0.5 to 3% of dental trauma in permanent dentition, but the majority of the sample, or 67.77%, is not aware of the correct procedure to follow in both preschool and school age, despite the fact that 16% have already heard of dental trauma and this is of concern as the probability of obtaining a favorable prognosis following treatment is significantly reduced. 15 16 17

In fact, having the possibility of carrying out an emergency maneuver at the scene of the accident could allow to obtain a restitutio ad integrum of the periodontal ligament and the functionality of the tooth. 6

In this case, it should be taken care of touching the tooth only from the coronal part and, if dirty, rinsing it for a maximum of 10 seconds under cold water without rubbing, as it would tear the fibers of the ligament, and then repositioning and stabilizing it using a cloth or a handkerchief. 6 12 13

If, on the other hand, this is not possible, a multifactorial evaluation of the “extra-alveolar persistence” will be fundamental, so we will talk about early reimplantation when the periodontal membrane is still viable and the time in which the tooth has remained outside the alveolus is a maximum of 45 to 60 minutes, up to a maximum of 24 hours if properly stored, while the term late reimplantation is used when the periodontal ligament is now necrotic, since the permanence of the element outside its seat was longer, the “conservation methods,” which involve using physiological saline solution, with a storage time of ∼120 minutes, or milk which, due to its composition and osmolarity, allows to maintain vitality for a few hours, or, if these are not available, the saliva inside the patient's oral cavity at the level of the gingival fornix 18 19 ; the “dental and alveolar conditions” for the presence or absence of fractures.

Therefore, previous knowledge about procedures and timing of intervention plays a fundamental role, as testified by 10% of the sample who, having already heard about dental trauma, in case of avulsion, would have known how to react. 5 6 10 12 13 20

Finally, in Tables 4 and 8 , we examine the question “Do you know that a soft diet is recommended in the 10/15 days following the trauma and, in younger children, the use of a pacifier is not recommended?,” as stated by the Ministry of Health. 13

In Table 1 , there are no significant differences related to the age of the subject and 57.98% of the parents claim to know this procedure.

In Table 8 , on the other hand, the fact that they have already heard of it previously has a positive effect on the knowledge of the sample, as 15.43% know which precautionary measures to adopt following a trauma.

The fact that there has been a generally positive response in relation to this question may derive from previous knowledge on the part of parents and teachers about other more common dental procedures that, however, require similar precautions. Especially with reference to the recommended diet, it is possible to transpose the information deriving from the execution of a filling as a treatment for a cavity or from an orthodontic treatment in the weeks following a dental trauma.

It is, therefore, noted that the more “common” a practice is, the more it is known by the population, while it is precisely in what happens more rarely but with important consequences that it is necessary to pay attention and carry out an information work

## Conclusion

The chances of developing dental trauma following an accidental event are real and can have considerable consequences from an aesthetic, functional, and economic point of view, with a compromise in the patient's quality of life.

Taking into consideration the most affected age groups, the work performed on the subjects plays a fundamental role, through the adoption of preventive measures depending on the context and the reduction in bad habits and attitudes at risk, and on who is responsible for them and lives in close contact with them on a daily basis, through a specific and targeted information and study activity.

Therefore, it emerges the need of spreading more awareness in this area, placing a spotlight on issues that, fortunately, are not addressed on a daily basis, but which, once they have occurred, require greater care by support figures, such as parents and teachers, and by health professionals.

Preserving the oral health of individuals in developmental age allows them to enjoy a state of well-being on a physical, but also psychic level, as it allows them to acquire personal safety and cultivate interpersonal relationships.
