# Supplementary material for: The Role of Information in Dental Traumatology in Patients during Developmental Age: A Cognitive Investigation
Source: Eur J Dent. 2021 Oct 22;16(2):296–301. doi: 10.1055/s-0041-1735789 (PMC9339915; doi:10.1055/s-0041-1735789)
Supplement: Supplementary file 1 — Supplementary Material [file 10-1055-s-0041-1735789-s2141541.pdf]

Appendix A: Questionnaire

Q1 Year of birth (of your child)
Open question
Q2 Gender (of your child)
Open question
Q3 Country of origin of the family
Open question
Q4 Have you ever heard of "dental trauma"
1. Yes
2. No

Q5 Has the child in question ever been subject to dental trauma?
1. Yes
2. No

Q6 Have you ever been told that scientific studies ascertain a greater risk of incurring dental trauma in children who already have a previous history?
1. Yes
2. No

Q7 Do you know that there can be an individual predisposition to trauma that depends on systemic conditions, particular anatomical structures, and defects in the calcification of the enamel?
1. Yes
2. No

Q8 Has it ever been explained to you that a complication at the level of deciduous ("milk") dentition could have consequences on the bony structures supporting both deciduous and permanent teeth?
1. Yes
2. No

Q9 If the formation of a tooth is not yet complete, can a trauma lead to the interruption of its vitality, with consequent "necrosis"?
1. Yes
2. No

Q10 Do you know that the frequency peaks are found around ⅔ years of age for the child's "walking" and around 8/10 years in conjunction with sports activities?
1. Yes
2. No

Q11 Which teeth will be most affected?
Open question
Q12 Are you aware of the fact that, in the event of a fracture and detachment of a dental fragment, it should NOT be dried with paper and stored in foil, but must be kept moistened to avoid necrosis of the tissue fibers?
1. Yes
2. No

Q13 Which of these liquids is not suitable for storage?
1. Water
2. Milk
3. Physiological solution

Q14 What is the maximum time limit within which to go to a dental office?
1. 6 h
2. 12 h
3. 24 h

Q15 If the tooth has completely come out of the bone (avulsion), do you know that you should try to re-implant the tooth in the socket or, if this is not possible, store it in milk or saline and go to the dentist within 60 minutes to have a favorable prognosis?
1. Yes
2. No

Q16 Are you aware of the fact that after a dental trauma it is necessary to adopt even more meticulous oral hygiene, using a soft bristle brush, to avoid the appearance of symptoms such as pain, swelling, mobility, and fistulas?
1. Yes
2. No

Q17 Do you know that in the 10/15 days following the trauma a soft diet is recommended and, in younger children, the use of a pacifier is recommended?
1. Yes
2. No

 © 2021. The Author(s).
